# Effects of Alcohol Consumption and Smoking on the Onset of Hypertension in a Long-Term Longitudinal Study in a Male Workers’ Cohort

**DOI:** 10.3390/ijerph182211781

**Published:** 2021-11-10

**Authors:** Tamotsu Nagao, Kazuhiro Nogawa, Koichi Sakata, Hideki Morimoto, Kotaro Morita, Yuka Watanabe, Yasushi Suwazono

**Affiliations:** Department of Occupational and Environmental Medicine, Graduate School of Medicine, Chiba University, Chiba 260-8670, Japan; nagao_tamotsu@khi.co.jp (T.N.); nogawa@chiba-u.jp (K.N.); sakata_ko@khi.co.jp (K.S.); morimoto@oh-handling.com (H.M.); morita_kotaro@khi.co.jp (K.M.); watanabe155@chiba-u.jp (Y.W.)

**Keywords:** alcohol consumption, smoking, hypertension, long-term longitudinal study, workers’ cohort, pooled logistic regression analysis

## Abstract

Aim: To determine the effects of alcohol consumption and smoking on the onset of hypertension in a long-term longitudinal study. Methods: 7511 non-hypertensive male workers were enrolled. This cohort study was performed over an 8-year period using the results of the annual workers-health screening. The end-point was defined as systolic blood pressure ≥ 140 mmHg, diastolic blood pressure ≥ 90 mmHg, or use of antihypertensive drugs. For alcohol consumption, weekly alcohol intake (g ethanol/week) was estimated (1 “gou” = 22 g ethanol). Annual survey data were analyzed by pooled logistic regression that included alcohol consumption, smoking, age, body mass index, job schedule types, habitual exercise, and blood test measurements into the statistical model. Results: A significant positive dose–response relationship between alcohol consumption and onset of hypertension was observed, with synergistic health effects present. Compared with abstainers and nonsmokers, the adjusted odds ratios (95% confidence interval) for the onset of hypertension were: 1.51 (1.27–1.79) for 154 g ethanol/week and nonsmokers, and 1.81 (1.54–2.11) for 154 g ethanol/week and smokers. An interaction between alcohol and smoking was confirmed. Conclusions: This study provided information useful to the prevention of hypertension. By reducing alcohol consumption and smoking simultaneously, the risk of hypertension may be considerably lowered.

## 1. Introduction

The Japanese Society of Hypertension Guidelines for the Management of Hypertension (JSH 2019) has estimated the number of hypertensives to be about 43 million, including only 12 million (27%) well-controlled ones [1,2,3,4]. Furthermore, approximately 50% of cardiovascular deaths are estimated to be due to blood pressure > 120/80 mmHg [1,2,5,6]. In another survey, the prevalence of hypertension (systolic blood pressure > 140 mmHg) was 29.9% for men and 24.9% for women, with significant decreases seen in both men and women over the last 10 years [7]. For the management of hypertension, six points for lifestyle modification are proposed: 1, Salt reduction; 2, Increased intake of vegetables/fruits; 3, Maintaining proper body weight; 4, Exercise therapy; 5, Reduction of alcohol intake; 6, Smoking cessation [1,8,9,10].

In this way, the onset of hypertension is affected by not just one factor but multiple factors, including both alcohol intake and smoking. In Japan, the prevalence of smoking in Japan in 2019 was 16.7% (27.1% for men and 7.6% for women), showing a largely downward trend year-by-year [7]. Regarding alcohol, the percentage of those drinking an amount sufficient to increase lifestyle risk (defined as a pure alcohol intake per day of 40 g ethanol for men and 20 g ethanol for women) was 14.9% for men and 9.1% for women, showing an almost flat trend in the last 10 years [7].

Many past studies identified alcohol as one factor that worsens hypertension [11,12]. In these studies, a dose–response relationship between alcohol consumption and hypertension was specifically noted. On the other hand, the relationship between smoking and hypertension was not found to be significant [13,14]. However, a transient increase in blood pressure while smoking cigarettes, as well as findings supportive of a causal association of smoking burden with a higher resting heart rate, were noted, despite how no direct relationship between smoking and hypertension has been documented [13,14]. Furthermore, numerous studies on the risks of cardiovascular disease (stroke and heart disease) have found that alcohol and smoking raise by several fold the risk of cardiovascular diseases [15,16]. In these studies, alcohol, smoking, and hypertension are all factors similarly affecting the outcome of cardiovascular diseases. Some previous studies have investigated the relationship between hypertension and both alcohol and smoking [17], but none, to our knowledge, have focused on the synergistic health effects of the two together on hypertension.

To help prevent cardiovascular diseases, it is important to reduce the risk of hypertension. Therefore, obtaining information on alcohol, smoking, and blood pressure is very important for the legally required health screening in the workplace in Japan [15,18,19].

Against this background, we first aimed in this study to determine the dose–response relationships between both alcohol consumption and smoking and the onset of hypertension. Next, we clarified whether synergistic health effects of alcohol consumption and smoking on the onset of hypertension exist.

## 2. Materials and Methods

### 2.1. Study Population

At annual workers-health screenings in a manufacturing company, this prospective cohort study of Japanese male workers was performed over an 8-year period from 2002 to 2010. The subject pool comprised 7511 male workers at a manufacturing company, out of a possible 10,900 persons who had participated in the first-year annual screening. Excluded at baseline were those who did not receive subsequent-year screening (1339 men), those who had already been diagnosed with hypertension (1491 men), and those with any missing data in the year of entry (559 men).

### 2.2. Measurements

In the annual workers-health screening, alcohol consumption and smoking were asked about and blood pressure was measured.

The end-point—the onset of hypertension—was defined as a systolic blood pressure (sBP) ≥ 140 mmHg or diastolic blood pressure (dBP) ≥ 90 mmHg or the start of antihypertensive drug treatment. About the use of any antihypertensive drugs, public health nurses and industrial physicians directly asked about this point at the time of the screening.

We asked the participants to fill in the questionnaire regarding drinking, the number of times he/she drank alcohol per month and the alcohol consumption per day. The question regarding alcohol consumption was “How many ‘gou’ do you drink in total per day? (1 gou of Japanese sake is equivalent to 500 mL of beer, 60 mL of whiskey, 180 mL of wine, or 110 mL of distilled spirits)”. Then, weekly alcohol intake (g ethanol/week) was calculated by multiplying the frequency of alcohol consumption by the quantity (gou/day × 22 g ethanol/gou). Then, we divided the intake into 5 categories: 0, 1–76, 77 (3.5 gou)–153, 154 (7.0 gou)–307, or 308 (14.0 gou) (g ethanol/week).

For smoking, we asked whether the subject was a smoker or not and how many cigarettes were smoked per day. Then, we divided the number of cigarettes smoked into 4 categories: 0, 1–10, 11–20, or 21 (cigarettes/day).

The number of groups for alcohol consumption and smoking were determined to clarify the dose–response relationships for these variables. Then those groups were combined to simplify the evaluation of the interaction (synergistic effect).

The other adopted covariates were age (years), body–mass index (BMI) (kg/m^2^), HbA1c (%), total serum cholesterol (TC) (mg/dL), aspartate aminotransferase (AST) (IU/L), uric acid (UA) (mg/dL), creatinine (Cre) (mg/dL), job schedule type, and habitual exercise. Job Schedule type was categorized into ‘Daytime’ or ‘2 or 3-shiftwork’. Habitual exercise was categorized into ‘Absence’, ‘once–twice/month’, ‘once–twice/week’ or ‘≥3 times/week’.

### 2.3. Statistical Analysis

Age, BMI, blood pressure, and alcohol consumption were summarized using arithmetic mean and standard deviation (SD). For blood test measurements, we assumed a lognormal distribution and summarized using geometric mean (GM) and geometric standard deviation (GSD). Number of cases, person–years of observation, and incidence rate for hypertension were summarized and grouped according to age.

The annual survey data were analyzed by pooled logistic regression analysis, and each 1-year examination interval was treated as a separate mini follow-up study [20]. Then, all these 1-year follow-ups were pooled and the odds ratios for the onset of hypertension were analyzed using a logistic regression model. In addition, alcohol consumption and smoking, or joint categories of both and other related factors such as age, BMI, job schedule type, habitual exercise, and blood test measurements (HbA1c, TC, AST, UA, and Cre), were included simultaneously in the statistical model. The odds ratios were corrected for fluctuations including calculation of the annual changes in each variable. For the blood test measurements, we performed a logarithmic transformation with 1.5 as the base. Therefore, odds ratios for a 1.5-fold increase in each item are shown in the results. Thereafter, we stratified the subjects into groups of over-50 and under-50 years old, and determined and examined the presence/absence of any age-dependent effects on the obtained OR. Furthermore, in order to verify the synergistic effect, the median and 95% CI of the relative excess risk due to the interaction (RERI), attributable proportion (AP), and synergy index (S) were calculated by the bootstrap method.

All analyses were performed using IBM SPSS 19J statistical software (IBM Business Analytics, Tokyo, Japan). *p* values < 0.05 were considered statistically significant.

## 3. Results

Table 1 summarizes the characteristics of the subjects at the study’s beginning year, grouped according to age. The mean age was 41.3 years. The mean blood pressure was increased with increasing age. Additionally, with increasing age the mean alcohol consumption increased, while the overall mean alcohol consumption was 110 g ethanol/week (=5.0 gou/week). With increasing age, nonsmokers and heavy smokers (21 cigarettes/day) both increased in number, while overall smoking prevalence was 57.9%.

Table 2 indicates the number of cases, person-years of observation, and incidence rate for hypertension—grouped according to age. According to the criteria adopted in this survey, 2351 persons (31.3%) showed an onset of hypertension. The onset of hypertension was observed more frequently with increasing age (11.9% vs. 48.2%, <29 years vs. 50–54 years).

The crude and adjusted odds ratios (aOR) and 95% confidence intervals (CI) for the onset of hypertension are shown in Table 3. The aOR were adjusted for the effect of all other covariates using pooled logistic regression.

**Figure 1:**
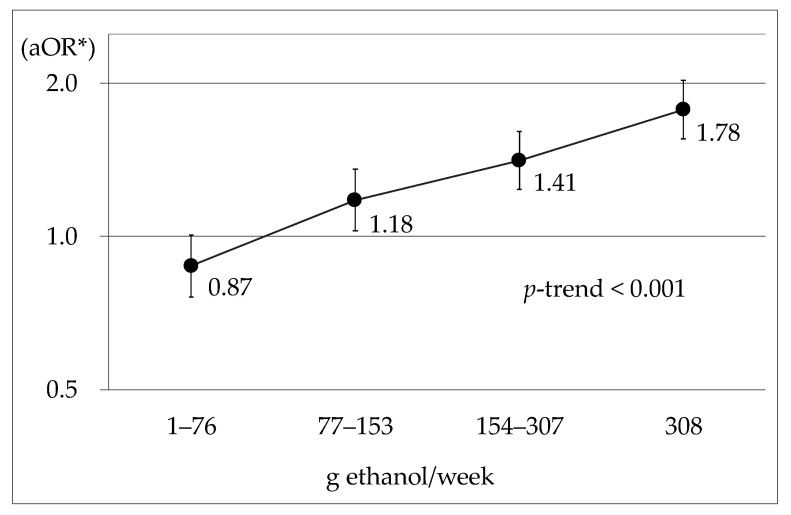
aOR of the onset of hypertension for weekly alcohol consumption compared with abstainers. * Smoking, age, BMI, job schedule type, habitual exercise, and blood test measurements (HbA1c, TC, AST, UA and Cre) were adjusted.

For alcohol consumption compared with abstainers (0 g ethanol/week), the aOR (95% CI) for other drinking groups were: 0.87 (0.76–1.01) (*p* = 0.060) for 1–76 g ethanol/week; 1.18 (1.02–1.35) (*p* = 0.022) for 77–153 g ethanol/week; 1.41 (1.24–1.61) (*p* < 0.001) for 154–307 g ethanol/week; and 1.78 (1.56–2.02) (*p* < 0.001) for 308 g ethanol/week. Figure 1 shows in graph form that there was a significant positive dose–response relationship between the onset of hypertension and alcohol consumption.

**Figure 2:**
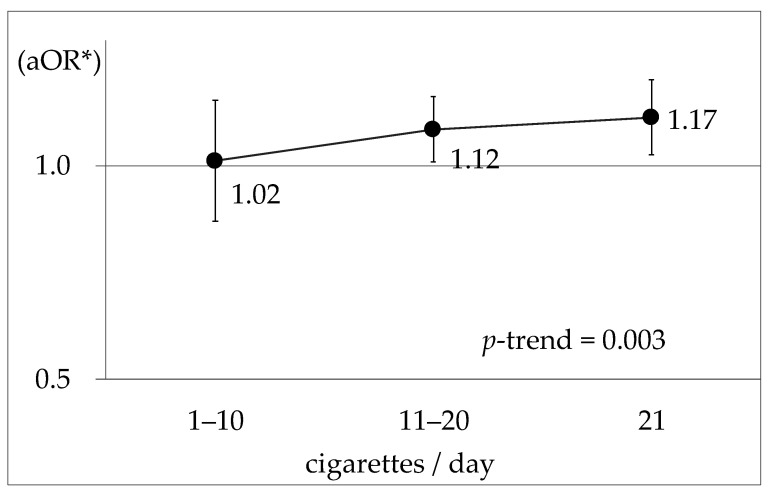
aOR of the onset of hypertension for tobacco consumption compared with nonsmokers. * Alcohol consumption, age, BMI, job schedule type, habitual exercise, and blood test measurements (HbA1c, TC, AST, UA and Cre) were adjusted.

For smoking compared with nonsmokers (0 cigarettes/day), the aOR (95% CI) for other smoking groups were: 1.02 (0.83–1.24) (*p* = 0.881) for 1–10 cigarettes/day; 1.12 (1.01–1.25) (*p* = 0.029) for 11–20 cigarettes/day; and 1.17 (1.03–1.32) (*p* = 0.012) for 21 cigarettes/day. Figure 2 shows in graph form that there was a positive dose–response relationship between the onset of hypertension and smoking.

Table 4 also showed the aOR (95% CI) for the onset of hypertension, but only regarding the synergistic effects of alcohol consumption and smoking. Compared with abstainers (0 g ethanol/week) and nonsmokers (0 cigarettes/day), the aOR (95% CI) for other drinking and smoking groups were: 1.12 (0.94–1.34) (*p* = 0.210) for abstainers and smokers; 1.01 (0.86–1.20) (*p* = 0.866) for 1–153 g ethanol/week and nonsmokers; 1.11 (0.93–1.31) (*p* = 0.240) for 1–153 g ethanol/week and smokers; 1.51 (1.27–1.79) (*p* < 0.001) for 154 g ethanol/week and nonsmokers; and 1.81 (1.54–2.11) (*p* < 0.001) for 154 g ethanol/week and smokers. Furthermore, we stratified the subjects into groups of over-50 and under-50 years old and determined the presence/absence of any age-dependent effects on obtained OR, but no significant difference was found.

Furthermore, in terms of the synergistic effects of drinking 154 g ethanol/week and smoking, the median (95% CI) for RERI was 0.19 (−0.06,0.43), AP was 0.11 (−0.03,0.24), and S was 1.32 (0.93,2.18). Although there was only a slight difference in the significance, the existence of some synergistic effect was suggested.

## 4. Discussion

This prospective cohort study in male workers at a manufacturing company investigated the synergistic health effects of alcohol consumption and smoking on the onset of hypertension. Summarizing the results, if alcohol consumption was under 154 g ethanol (7.0 gou)/week, the adjusted odds ratios (aOR) of either smokers or nonsmokers was about 1.0–1.1, which is considered not significant. However, if alcohol consumption was over 154 g ethanol (7.0 gou)/week, the aOR of nonsmokers was over 1.5 and the aOR of smokers was over 1.8, and they were statistically significant compared to abstainers and nonsmokers. Consequently, synergistic health effects of alcohol consumption and smoking on the onset of hypertension were documented. Furthermore, a correct understanding of these relationships can especially help with health guidance regarding alcohol and smoking in workers based on accurate evidence. Regarding these results and with reference to those of previous studies, several points were considered noteworthy and listed below.

### 4.1. A Positive Dose–Response Relationships between Alcohol Consumption and the Onset of Hypertension

The effects of alcohol on the human body are diverse. The underlying mechanisms of the blood pressure rise due to alcohol drinking include an increased constriction response of blood vessels, hyperactivity of the sympathetic nervous system that accelerates the beating of the heart, and loss of magnesium and potassium from the kidneys. In past epidemiological studies, a large percentage (34.5%) of hypertension was alcohol-induced in a Japanese male population [21]. There was a significant dose–response relationship between the amount of alcohol consumed and the odds ratio for hypertension, in that an alcohol drinking habits of more than 154 g ethanol/week had been shown to exacerbate high blood pressure. Moreover, the degree of exacerbation increased as the volume of alcohol consumption increased to 308 g ethanol/week and 462 g ethanol/week. On the other hand, a reduction in alcohol intake was associated with increased blood pressure reduction [11,12]. The reduction in blood pressure was especially marked in those participants who consumed six or more drinks per day if they then reduced their intake by 50%, which means that reducing alcohol intake lowers blood pressure in a dose-dependent manner with an apparent threshold effect [12]. Other studies showed that not only the daily consumption of alcohol but also the habit of drinking alcohol more than 5 days/week increased the risk of the onset of hypertension [22]. Furthermore, a meta-analysis from a total of 12 cohort studies showed that a linear dose–response (a relative risk of 1.57 at 50 g ethanol/day, and 2.41 at 100 g ethanol/day) was seen for men [23].

In this study, we conducted a pooled logistic regression analysis based on a large-scale cohort study that defined the end-point as the onset of hypertension, and we believed the results establish a positive dose–response relationship between alcohol consumption and the onset of hypertension. Although this result was similar to those from some past studies, we consider the accuracy and reliability of the present study to be a considerable improvement due to the larger sample size, the longer observation period of 8 years, and the statistical method used, compared to those in past studies.

### 4.2. A Positive Dose–Response Relationships between Smoking and the Onset of Hypertension

Some observations suggest a transient increase while smoking a cigarette and that smoking increases blood pressure for more than 15 min [24]. Nicotine in tobacco smoke is said to play a major role in the mechanism of transient increase in blood pressure, and nicotine stimulates the adrenal glands to releases catecholamines and stimulate the sympathetic nervous system, thereby causing constriction of peripheral blood vessels, increase in blood pressure, and increase in heart rate [25]. On the other hand, although there are few reports of smoking as a direct cause of the onset of persistent hypertension, there are some about the risk of developing cardiovascular diseases (stroke and heart diseases), and as such the combined action of smoking and hypertension is thought to increase the incidence of cardiovascular problems [15]. However, few reports have associated smoking with the onset or exacerbation of hypertension or documented any clear evidence of a relationship between smoking and hypertension itself [13,14].

In this study, for the same reasons as for alcohol, we believed the results established a positive dose–response relationship between smoking and the onset of hypertension. However, the increase in the odds ratio was found to be gradual compared to that of alcohol consumption, so that as a single factor, alcohol consumption has a greater impact on the onset of hypertension than smoking.

### 4.3. Synergistic Health Effects of Alcohol Consumption and Smoking on the Onset of Hypertension

Based on the above points, both alcohol and smoking share a common mechanism for the onset of hypertension as a single factor in that they both stimulate the sympathetic nervous system. However, as mentioned above, the factors that act on the sympathetic nervous system are different, and there is both a transient increase in blood pressure and the onset of persistent hypertension. In this way, considering that alcohol and smoking are combined and that the sympathetic nervous system is frequently, repeatedly stimulated in such a way as to promote the onset of hypertension, we thought—from a combined perspective—that alcohol and smoking are likely to work synergistically to promote the onset of hypertension. Regarding the combined effect of alcohol and smoking in past studies, one study found that achievement of the treatment goal was reduced when alcohol consumption and smoking were continued even during the hypertension treatment period [26]. However, as far as we know, no study has focused on the combined effects of alcohol and smoking from the perspective of developing hypertension from the non-hypertensive state. On the other hand, as an example of how alcohol and smoking act synergistically in the case of diseases other than hypertension, one study showed that the consumption of alcohol and smoking was positively correlated with development of oral squamous cell carcinoma, with an odds ratio of 5.37 (95% CI: 3.54–8.14) [27]. Similarly, the synergistic effects of alcohol and smoking are considered to play a major role in other various cancers as well.

The present study showed that, in the group with low alcohol consumption, there was no significant difference in the onset of hypertension according to the amount smoked; whereas in the group with high alcohol consumption, there was a significant difference in the onset of hypertension even if the amount of smoking was small. Furthermore, when the amount of smoking increased, the risk of developing hypertension increased synergistically. In this way, the following relationships were concluded to be inter-related: between the greater impact of alcohol consumption and the positive dose–response relationship of the onset of hypertension; and between the weaker but less influential number of cigarettes smoked and the positive dose–response relationship of the onset of hypertension. Therefore, this can be considered to be a synergistic health effect due to the combination of alcohol consumption and smoking.

### 4.4. Preventing the Onset of Hypertension, and Health Guidance about Alcohol and Smoking

Of course, it is important to implement optimal health-care management for workers at the onset of hypertension, and more importantly to prevent the onset of hypertension from the perspective of preventive medicine. Alcohol and cigarettes are particular discretionary lifestyle items that are apt to be enjoyed by workers at a manufacturing company, and alcohol and smoking indulgence may be increasing in this population. Meanwhile, it is important to implement health guidance about alcohol and smoking, not only to prevent the onset of hypertension but also about other various lifestyle-related diseases, including cancers [28].

Health guidance is often implemented as a follow-up measure after annual workers-health screening. In addition, it may be provided at any time for health consultation within the company, but even in this case guidance is given based on the results of the latest screening. Therefore, at the annual workers-health screening, it is necessary that the amount of alcohol consumed and the amount of smoking be recorded in an interview, and that it is converted into data such that changes over time can always be confirmed. This would be useful for both the subject and the health-care provider. since this would enable clear visualization of the alcohol and smoking habits. In other words, the guidance effect might be enhanced by providing health guidance with visualization of the serial changes of the data.

Furthermore, based on the results of this study, it is considered that more optimal health guidance can be given if the subject can be motivated to moderate alcohol consumption and quit smoking by adding this kind of objective explanation.

On the other hand, we have also considered the implications of the present findings in the wider context of general populations other than workers involved in the health-screening program. In Japan, apart from annual health screenings for workers, there is an initiative from the government side in which persons aged 40–74 years (including non-workers), who are also enrolled in the National Health Insurance Program, are eligible for Lifestyle Health Check-ups and Lifestyle Health Guidance. Similarly, since the Lifestyle Health Check-ups also include interviews regarding alcohol consumption and smoking and blood pressure measurements, we considered that the Lifestyle Health Guidance could be handled in the same way in this study. Therefore, it is also necessary to consider the aspects of public health information dissemination, such as recommending regular health checks and cautioning against drinking and smoking to prevent the onset of hypertension.

### 4.5. Limitations

First, the outcome may seem incidental, meaning that the aOR might overestimate the associations. Previously, a difficulty occurred when interpreting OR as the risk, when the incidence was more than 10% and the obtained OR was more than 2.5 [29]. In the present study, the incidence of hypertension based on person-years was 9.03% and obtained ORs were less than 2.5, indicating that any bias due to the OR was less likely in the present results.

One limitation of this study was that the end-point of the onset of hypertension was defined as whether, in the annual workers-health screening, systolic blood pressure (sBP) ≥ 140 mmHg or diastolic blood pressure (dBP) ≥ 90 mmHg was present; the end-point also included how the start of workers taking antihypertensive drugs during the one year since the last annual workers-health screening was confirmed by the annual workers-health screening. Therefore, if a disease that causes secondary hypertension (such as primary aldosteronism, etc.) had developed rapidly during the past year, the causal relationship between alcohol and smoking and the onset of hypertension might have been obscured. It is estimated that 5–10% of hypertensive patients have secondary hypertension [30]. If appropriately diagnosed and treated, patients with a secondary hypertension might be cured or at least show an improvement in their blood pressure control; whereas, as so-called resistant hypertension, there are many cases in which blood pressure cannot be controlled well only by antihypertensive drugs or lifestyle modification [31]. Therefore, in secondary hypertension, it is difficult to prevent the onset of hypertension due to alcohol and smoking.

Another limitation was that it would be very important to consider pack-years, or other life-long smoking information, rather than a punctual estimate of smoking in the analysis to associate with hypertension. However, this survey did not collect information on the number of years of smoking at the beginning of the survey, and so this parameter could not be evaluated.

Furthermore, another limitation of this study was the possibility that it might not have accurately analyzed alcohol consumption and smoking due to some inconsistent data; for example, some persons may have continued to use the same amount of alcohol and number of cigarettes every year for many years, whereas others may have previously smoked but have now stopped. However, the use of a pooled logistic regression analysis in this study is one of its strengths, and this analysis has an advantage compared with usual Cox proportional hazards. In general Cox proportional hazards, a factor can be analyzed only at the time of entry. On the other hand, pooled logistic regression analysis can reflect annual survey data and take into account changes in job schedule type, habitual exercise, and blood test measurements [20]. Therefore, we believe that the present results are more accurate than those of other past studies.

A further limitation is the phenomenon of “sick-quitters”. Participants with high blood pressure from the beginning were excluded. However, it is true that some participants may have stopped drinking due to a medical condition other than hypertension, but were registered as participants because, at the time, they did not meet the criteria for high blood pressure. In such a case, it was possible that even if they had stopped drinking by then, it may have affected the onset of hypertension due to the effects of the previous heavy drinking. If they were “sick-quitters” within the observation period with a subsequent new onset of hypertension, they could have been considered in the pooled logistic regression analysis. On the other hand, if they were “sick-quitters” before the observation period with the new onset of hypertension occurring within the observation period, it might be impossible to distinguish this from being caused by aging or other factors.

Moreover, although the obtained data were self-reported, the contents of the questionnaire that were filled in before the screening can be confirmed directly with the person himself by the interviewing public health nurse and the medical examination doctor during the screening. Of course, there may be some inaccurate self-reporters, but from the system described above the number of such inaccurate self-reporters seems to not be high. The area of the target company is a rural one, and there are many blue-collar workers. Therefore, drinking and smoking are not considered to be very unfavorable behaviors by the subjects themselves, and their thinking is comparable to that of the other inhabitants of the same rural area, resulting in less fear of stigma. We think that this factor cannot be denied as a potential bias, but the impact would not be that great.

Finally, we focus on the generalizability of the present study to the general population. Since this study used samples limited to the steel industry, men, and workers, generalizability may be limited. However, from the perspective of the effects of drinking and smoking on the physiological mechanism for the onset of hypertension, some degree of generalizability is possible in theory. To resolve this in the future, similar studies will be needed focusing on various other general populations, such as those in industries other than the steel industry, office workers, women, and non-workers, to further clarify the possible generalizability of the present results.

There are limitations to the above several points. However, many occupational health physicians and corporate workers are aware that lifestyle-related habits such as alcohol drinking and smoking will worsen high blood pressure; this study documents that the onset of hypertension has a dose–response relationship depending on the amount of both alcohol consumption and smoking, with the two showing a synergistic health effect. Therefore, in health guidance and from the perspective of prevention, we hope that the present results will promote recognition of the role of these behaviors in the onset of hypertension, and thereby be able to help trigger the necessary behavioral changes to prevent it.

## 5. Conclusions

The present study identified significant positive dose–response relationships between alcohol consumption and the onset of hypertension, as well as between smoking and the onset of hypertension. In addition, there were synergistic health effects of alcohol consumption and smoking on the onset of hypertension. By reducing alcohol consumption and smoking simultaneously, the risk of hypertension may be considerably lowered.

## Figures and Tables

**Table 1:** Characteristics of the subjects at study entry year, grouped according to age.

	Age Group (yrs)				
	≤29	30–39	40–49	≥50	Total
	Mean (SD)	Mean (SD)	Mean (SD)	Mean (SD)	Mean (SD)
Age (years)	24.9 (3.2)	33.9 (2.9)	45.2 (2.8)	54.7 (3.9)	41.3 (12.2)
Body mass index (kg/m^2^)	22.9 (3.5)	23.6 (3.3)	23.8 (2.8)	23.6 (2.7)	23.5 (3.1)
Systolic blood pressure (mmHg)	123.1 (9.9)	124.2 (9.6)	125.7 (9.3)	127.3 (9.0)	125.3 (9.5)
Diastolic blood pressure (mmHg)	72.5 (7.6)	75.3 (7.4)	77.8 (6.9)	78.2 (6.6)	76.2 (7.5)
Mean blood pressure (mmHg)	89.4 (7.7)	91.6 (7.5)	93.8 (7.1)	94.6 (6.7)	92.6 (7.5)
Alcohol consumption (g ethanol/week)	52.8 (4.0)	92.4 (5.2)	140.8 (6.2)	138.6 (6.3)	110 (5.8)
	GM (GSD)	GM (GSD)	GM (GSD)	GM (GSD)	GM (GSD)
HbA1c (%)	4.8 (1.1)	4.9 (1.1)	5.1 (1.1)	5.3 (1.1)	5.0 (1.1)
Total serum cholesterol (mg/dL)	171.8 (1.2)	191.1 (1.2)	199.9 (1.2)	201.1 (1.2)	191.5 (1.2)
Aspartate aminotransferase (IU/L)	18.8 (1.4)	20.2 (1.4)	20.4 (1.4)	21.1 (1.4)	20.2 (1.4)
Creatinine (mg/dL)	0.80 (1.1)	0.80 (1.1)	0.79 (1.2)	0.80 (1.2)	0.80 (1.1)
Uric acid (mg/dL)	5.7 (1.2)	5.8 (1.2)	5.7 (1.3)	5.6 (1.3)	5.7 (1.3)
	N (%)	N (%)	N (%)	N (%)	N (%)
Job Schedule type					
Daytime	881 (50.3%)	984 (63.0%)	1138 (66.1%)	1635 (66.1%)	4638 (61.7%)
2 or 3-shiftwork	871 (49.7%)	578 (37.0%)	584 (33.9%)	840 (33.9%)	2873 (38.3%)
Smoking					
Nonsmokers	676 (38.6%)	593 (38.0%)	692 (40.2%)	1199 (48.4%)	3160 (42.1%)
1–10 cigarettes/day	222 (12.7%)	140 (9.0%)	80 (4.6%)	118 (4.8%)	560 (7.5%)
11–20 cigarettes/day	666 (38.0%)	577 (36.9%)	554 (32.2%)	661 (26.7%)	2458 (32.7%)
21 cigarettes/day	188 (10.7%)	252 (16.1%)	396 (23.0%)	497 (20.1%)	1333 (17.7%)
Habitual exercise					
Absence	646 (36.9%)	689 (44.1%)	789 (45.8%)	1038 (41.9%)	3162 (42.1%)
once-twice/month	320 (18.3%)	255 (16.3%)	241 (14.0%)	231 (9.3%)	1047 (13.9%)
once-twice/week	508 (29.0%)	439 (28.1%)	473 (27.5%)	687 (27.8%)	2107 (28.1%)
≥3 times/week	278 (15.9%)	179 (11.5%)	219 (12.7%)	519 (21.0%)	1195 (15.9%)

SD: Standard deviation. GM: Geometric mean. GSD: Geometric standard deviation.

**Table 2:** Number of cases, person-years of observation and incidence rate for hypertension grouped according to age.

	Age Group (yrs)					
	≤29	30–39	40–49	50–54	≥55	Total
Cases (%)	208 (11.9)	336 (21.5)	703 (40.8)	633 (48.2)	471 (40.5)	2351 (31.3)
Total person-years of observation	5167	5639	7199	5185	2853	26,043
Incidence rate per 1000 person years	40.3	59.6	97.7	122.1	165.1	90.3
Mean observed years per person	2.95	3.61	4.18	3.95	2.46	3.47

**Table 3:** Crude and adjusted odds ratios and 95% confidence intervals for the onset of hypertension.

Independent Variables	Crude OR	*p*	aOR * (95% CI **)	*p*
Weekly alcohol consumption compared with abstainers				
1–76 g ethanol (0.1–3.4 gou)	0.84 (0.73, 0.96)	0.010	0.87 (0.76, 1.01)	0.060
77–153 g ethanol (3.5–6.9 gou)	1.24 (1.08, 1.42)	0.002	1.18 (1.02, 1.35)	0.022
154–307 g ethanol (7.0–13.9 gou)	1.61 (1.42, 1.83)	<0.001	1.41 (1.24, 1.61)	<0.001
308 g ethanol (14.0 gou)	2.14 (1.89, 2.43)	<0.001	1.78 (1.56, 2.03)	<0.001
Tobacco consumption compared with nonsmokers				
1–10 cigarettes/day	0.80 (0.66, 0.97)	0.021	1.02 (0.83, 1.24)	0.881
11–20 cigarettes/day	1.00 (0.90, 1.10)	0.936	1.12 (1.01, 1.25)	0.029
21 cigarettes/day	1.29 (1.15, 1.44)	<0.001	1.17 (1.03, 1.32)	0.012

* adjusted odds ratio: odds ratios adjusted for the effect of all other covariates using pooled logistic regression. ** 95% confidence interval.

**Table 4:** Adjusted Odds ratios and 95% confidence intervals for the onset of hypertension associated with the synergistic effects of alcohol consumption and smoking.

Independent Variables	aOR (95% CI)	*p*
Alcohol and tobacco compared with abstainers and nonsmokers		
Abstainers and smokers	1.12 (0.94, 1.34)	0.210
1–153 g ethanol(0.1–6.9 gou) and nonsmokers	1.01 (0.86, 1.20)	0.866
1–153 g ethanol(0.1–6.9 gou) and smokers	1.11 (0.93, 1.31)	0.240
154 g ethanol(7.0 gou) and nonsmokers	1.51 (1.27, 1.79)	<0.001
154 g ethanol(7.0 gou) and smokers	1.81 (1.54, 2.11)	<0.001

## Data Availability

The data that support the findings of this study are available from the corresponding authors upon reasonable request.

## References

[1] Umemura S., Arima H., Arima S., Asayama K., Dohi Y., Hirooka Y., Horio T., Hoshide S., Ikeda S., Ishimitsu T. (2019). The Japanese Society of Hypertension Guidelines for the Management of Hypertension (JSH 2019). Hypertens. Res..

[2] Maruhashi T., Kihara Y., Higashi Y. (2020). Perspectives on the management of hypertension in Japan. Expert Opin. Pharmacother..

[3] Miura K., Nagai M., Ohkubo T. (2013). Epidemiology of hypertension in Japan: Where are we now?. Circ. J..

[4] Asakura E., Ademi Z., Liew D., Zomer E. (2021). Productivity burden of hypertension in Japan. Hypertens. Res..

[5] Fujiyoshi A., Ohkubo T., Miura K., Murakami Y., Nagasawa S.-Y., Okamura T., Ueshima H., for the Evidence for Cardiovascular Prevention from Observational Cohorts in Japan (EPOCH-JAPAN) Research Group (2012). Blood pressure categories and long-term risk of cardiovascular disease according to age group in Japanese men and women. Hypertens. Res..

[6] Kokubo Y., Matsumoto C. (2016). Hypertension Is a Risk Factor for Several Types of Heart Disease: Review of Prospective Studies. Adv. Exp. Med. Biol..

[7] The Ministry of Health, Labour, and Welfare The Results of the 2019 National Health and Nutrition Survey in Japan. https://www.mhlw.go.jp/content/10900000/000687163.pdf.

[8] O’Dickinson H., Mason J., Nicolson D.J., Campbell F., Beyer F.R., Cook J.V., Williams B., Ford G.A. (2006). Lifestyle interventions to reduce raised blood pressure: A systematic review of randomized controlled trials. J. Hypertens..

[9] Valenzuela P.L., Carrera-Bastos P., Gálvez B.G., Ruiz-Hurtado G., Ordovas J.M., Ruilope L.M., Lucia A. (2021). Lifestyle interventions for the prevention and treatment of hypertension. Nat. Rev. Cardiol..

[10] Ozemek C., Tiwari S., Sabbahi A., Carbone S., Lavie C.J. (2020). Impact of therapeutic lifestyle changes in resistant hypertension. Prog. Cardiovasc. Dis..

[11] Miller P.M., Anton R.F., Egan B.M., Basile J., Nguyen S.A. (2005). Excessive Alcohol Consumption and Hypertension: Clinical Implications of Current Research. J. Clin. Hypertens..

[12] Roerecke M., Kaczorowski J., Tobe S.W., Gmel G., Hasan O.S.M., Rehm J. (2017). The effect of a reduction in alcohol consumption on blood pressure: A systematic review and meta-analysis. Lancet Public Health.

[13] Linneberg A., Jacobsen R.K., Skaaby T., Taylor A.E., Fluharty M.E., Jeppesen J.L., Bjorngaard J.H., Åsvold B.O., Gabrielsen M.E., Campbell A. (2015). Effect of Smoking on Blood Pressure and Resting Heart Rate: A Mendelian Randomization Meta-Analysis in the CARTA Consortium. Circ. Cardiovasc. Genet..

[14] Leone A. (2011). Smoking and hypertension: Independent or additive effects to determining vascular damage?. Curr. Vasc. Pharmacol..

[15] Landini L., Leone A. (2011). Smoking and hypertension: Effects on clinical, biochemical and pathological variables due to isolated or combined action on cardiovascular system. Curr. Pharm. Des..

[16] Sleight P. (1993). Smoking and hypertension. Clin. Exp. Hypertens..

[17] Jacobs A., Pieters M., Schutte A.E. (2020). The association of PAI-1 with 24 h blood pressure in young healthy adults is influenced by smoking and alcohol use: The African-PREDICT study. Nutr. Metab. Cardiovasc. Dis..

[18] Kawano Y., Abe H., Takishita S., Omae T. (1998). Effects of alcohol restriction on 24-hour ambulatory blood pressure in japanese men with hypertension. Am. J. Med..

[19] Minami J., Ishimitsu T., Matsuoka H. (1999). Effects of Smoking Cessation on Blood Pressure and Heart Rate Variability in Habitual Smokers. Hypertension.

[20] D’Agostino R.B., Lee M.-L., Belanger A.J., Cupples L.A., Anderson K., Kannel W.B. (1990). Relation of pooled logistic regression to time dependent cox regression analysis: The framingham heart study. Stat. Med..

[21] Nakamura K., Okamura T., Hayakawa T., Hozawa A., Kadowaki T., Murakami Y., Kita Y., Okayama A., Ueshima H. (2007). The Proportion of Individuals with Alcohol-Induced Hypertension among Total Hypertensives in a General Japanese Population: NIPPON DATA90. Hypertens. Res..

[22] Núñez-Córdoba J.M., Martínez-González M.A., Bes-Rastrollo M., Toledo E., Beunza J.J., Alonso A. (2009). Alcohol consumption and the incidence of hypertension in a Mediterranean cohort: The SUN study. Rev. Española Cardiol..

[23] Taylor B., Irving H.M., Baliunas D., Roerecke M., Patra J., Mohapatra S., Rehm J. (2009). Alcohol and hypertension: Gender differences in dose-response relationships determined through systematic review and meta-analysis. Addiction.

[24] Groppelli A., Giorgi D.M.A., Omboni S., Parati G., Mancia G. (1992). Persistent blood pressure increase induced by heavy smoking. J. Hypertens..

[25] Perkins K.A., Epstein L.H., Jennings J.R., Stiller R. (1986). The cardiovascular effects of nicotine during stress. Psychopharmacology.

[26] Kékes E., Paksy A., Baracsi-Botos V., Szőke V.B., Járai Z. (2020). The combined effect of regular alcohol consumption and smoking on blood pressure and on the achievement of blood pressure target values in treated hypertensive patients. Orv. Hetil..

[27] Mello F.W., Melo G., Pasetto J.J., Silva C.A.B., Warnakulasuriya S., Rivero E. (2019). The synergistic effect of tobacco and alcohol consumption on oral squamous cell carcinoma: A systematic review and meta-analysis. Clin. Oral Investig..

[28] Mons U., Gredner T., Behrens G., Stock C., Brenner H. (2018). Cancers Due to Smoking and High Alcohol Consumption. Dtsch. Aerzteblatt Online.

[29] Zhang J., Yu K.F. (1998). What’s the relative risk? A method of correcting the odds ratio in cohort studies of common outcomes. JAMA.

[30] Tziomalos K. (2020). Secondary Hypertension: Novel Insights. Curr. Hypertens. Rev..

[31] Ott C., Schneider M.P., Schmieder R.E. (2013). Ruling out secondary causes of hypertension. EuroIntervention.

